# Drug Repurposing for Paracoccidioidomycosis Through a Computational Chemogenomics Framework

**DOI:** 10.3389/fmicb.2019.01301

**Published:** 2019-06-12

**Authors:** Amanda Alves de Oliveira, Bruno Junior Neves, Lívia do Carmo Silva, Célia Maria de Almeida Soares, Carolina Horta Andrade, Maristela Pereira

**Affiliations:** ^1^Laboratório de Biologia Molecular, Universidade Federal de Goiás, Goiânia, Brazil; ^2^Laboratório de Cheminformática, Centro Universitário de Anápolis, UniEVANGÉLICA, Anápolis, Brazil; ^3^Laboratório de Modelagem Molecular e Design de Medicamentos, Faculdade de Farmácia, Universidade Federal de Goiás, Goiânia, Brazil

**Keywords:** *Paracoccidioides* species, drug repurposing, genome-wide alignment, gene essentiality, molecular docking, *in vitro* assays

## Abstract

Paracoccidioidomycosis (PCM) is the most prevalent endemic mycosis in Latin America. The disease is caused by fungi of the genus *Paracoccidioides* and mainly affects low-income rural workers after inhalation of fungal conidia suspended in the air. The current arsenal of chemotherapeutic agents requires long-term administration protocols. In addition, chemotherapy is related to a significantly increased frequency of disease relapse, high toxicity, and incomplete elimination of the fungus. Due to the limitations of current anti-PCM drugs, we developed a computational drug repurposing-chemogenomics approach to identify approved drugs or drug candidates in clinical trials with anti-PCM activity. In contrast to the one-drug-one-target paradigm, our chemogenomics approach attempts to predict interactions between drugs, and *Paracoccidioides* protein targets. To achieve this goal, we designed a workflow with the following steps: (a) compilation and preparation of *Paracoccidioides* spp. genome data; (b) identification of orthologous proteins among the isolates; (c) identification of homologous proteins in publicly available drug-target databases; (d) selection of *Paracoccidioides* essential targets using validated genes from *Saccharomyces cerevisiae*; (e) homology modeling and molecular docking studies; and (f) experimental validation of selected candidates. We prioritized 14 compounds. Two antineoplastic drug candidates (vistusertib and BGT-226) predicted to be inhibitors of phosphatidylinositol 3-kinase TOR2 showed antifungal activity at low micromolar concentrations (<10 μM). Four antifungal azole drugs (bifonazole, luliconazole, butoconazole, and sertaconazole) showed antifungal activity at low nanomolar concentrations, validating our methodology. The results suggest our strategy for predicting new anti-PCM drugs is useful. Finally, we could recommend hit-to-lead optimization studies to improve potency and selectivity, as well as pharmaceutical formulations to improve oral bioavailability of the antifungal azoles identified.

## Introduction

Paracoccidioidomycosis (PCM) is a systemic mycosis caused by the saprobic and dimorphic *Paracoccidioides* species (Shikanai-Yasuda et al., 2017). Though a rare disorder from a global perspective, PCM is the most prevalent endemic mycosis in Latin America (Queiroz-Telles et al., 2017). Recent studies have shown that PCM is responsible for approximately half of deaths caused by systemic mycoses in Brazil (Martinez, 2017). Natural infection mainly affects low-income rural workers after inhalation of fungal conidia. The conidia transform into the pathogenic yeast in the lungs, triggering inflammatory responses, and formation of granulomatous lesions. The disease affects other tissues and organs, such as oral mucous membranes and skin. Consequently, this disease has negative social and economic impacts, especially in individuals in their most productive phase of life (Shikanai-Yasuda et al., 2017).

Anti-PCM chemotherapy requires long-term treatment and the current arsenal of chemotherapeutic agents is restricted to sulfamethoxazole-trimethoprim, itraconazole, and amphotericin B. However, several problems are associated with the use of these drugs, including high toxicity and incomplete elimination of the fungus (Shikanai-Yasuda, 2015). The discovery of new anti-PCM drugs with efficacy and fewer side effects is urgently needed.

Despite the need to discover and develop new antifungal drugs, the pharmaceutical industry under invests in this area, mostly because of the financial costs and risks of innovation for treatment of this disease of resource-poor countries. To overcome these limitations, drug repositioning may provide a promising strategy to find novel antifungal indications among approved drugs, or drug candidates in clinical trials (Aubé, 2012). This strategy is appealing because the drugs identified can avoid some early stages of drug discovery and development as their safety and pharmacokinetic profiles are already known. Consequently, drug repurposing can truncate the initial 6 years typically required for the conception of new chemical by entities, entering preclinical testing, or clinical trials directly (Novac, 2013; Jin and Wong, 2014). As such, drug repurposing could reduce costs, risks, and timelines to the market, and consequently provide strategic advantage in identifying new treatments of PCM (Ashburn and Thor, 2004; Hurle et al., 2013).

**FIGURE 1:**
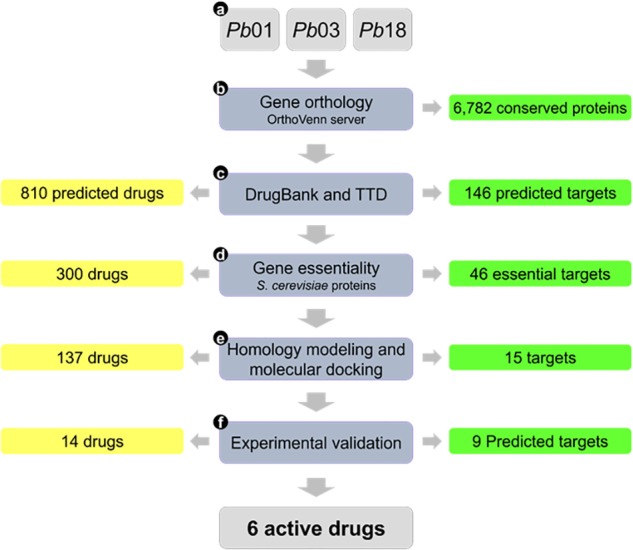
Flowchart summarizing the main steps of the study and corresponding results.

With genome and transcriptome data available for several *Paracoccidioides* spp. isolates, we have used a computational chemogenomics approach to repurpose new drugs for PCM. Chemogenomics is a powerful strategy that involves systematic identification of potential ligands based on the entire *Paracoccidioides* genome (Bredel and Jacoby, 2004; Andrade et al., 2018). Computational chemogenomics approach developed by our group presumes that proteins sharing enough similarity (homology) have enhanced the probability of sharing the same ligands (Andrade et al., 2018). In this work, we applied a computational chemo genomics framework based on innovative computational methods to predict new drugs with activity against *Paracoccidioides* spp. The approach uses the following steps (see Figure 1): (a) compilation and preparation of *Paracoccidioides* spp. genome data; (b) identification of orthologous proteins among genome isolates; (c) identification of homologous proteins in publicly available drug-target databases; (d) prediction of *Paracoccidioides* targets essentiality using genes of *Saccharomyces cerevisiae*; (e) homology modeling and molecular docking of the predicted targets and associated drugs; and (f) *in vitro* experimental validation of the top predicted drugs.

## Materials and Methods

### Computational Procedures

#### Mining of *Paracoccidioides* spp. Genomes

A list of all *Paracoccidioides lutzii* (*Pb*01), *Paracoccidioides americana* (*Pb*03), and *Paracoccidioides brasiliensis* (*Pb*18) proteins from the Broad Institute (Broad Institute, 2018) was compiled and duplicates removed. Orthologous proteins among the three genome isolates were identified using the OrthoVenn server (Wang et al., 2015). Pairwise sequence similarities between all input protein sequences were calculated with an expectation value (*e*-value) cut-off of >10^−20^. The *e*-value is the expected number of times a homology match will occur at random in a given set of trials (Neves et al., 2015).

#### Repurposing of Putative Drugs From Public Databases

Putative anti-PCM drugs were screened assuming that homologous proteins have enhanced the probability of sharing the same ligands (Andrade et al., 2018). A sequence-based similarity search was performed between *Pb*01 proteins and all drug targets available on DrugBank (Law et al., 2014) and the therapeutic targets database (TTD) (Li et al., 2018). The latest release of DrugBank contains 5,842 drug candidates and 2,556 approved drugs. TTD currently contains 9,528 drug candidates and 2,071 approved drugs. The chemical duplicates in the two databases were identified during virtual screening. These publicly available databases provide information about the known and explored therapeutic protein and nucleic acid targets, the targeted disease, pathway information, and the corresponding drugs interacting with each of these targets.

#### Computational Prediction of Essentiality

Essential genes, knockouts of which result in cell inviability or lethality, are important to the study of biological system robustness and effective drug target identification. Tools in essential genes of *S. cerevisiae* (model organism) were retrieved from the Database of Essential Genes (DEG) (Zhang, 2004; Zhang and Lin, 2009), in order to compare with prioritized *Pb*01 targets using OrthoVenn. The essentiality of the prioritized proteins was inferred by selecting *Pb*01 targets orthologous (*e*-value ≤ 10^−20^) to *S. cerevisiae* proteins experimentally determined to be essential.

#### Homology Modeling

The 3D structures of the predicted *Pb*01 targets were built using the SWISS-MODEL server (Bordoli et al., 2009; Biasini et al., 2014). Homology models were built using four main steps: (a) identification of structural templates in Protein Data Bank (PDB) (Rose et al., 2015); (b) alignment of protein sequences and template structures; (c) model building; and (d) analysis of the geometrical and stereochemical quality of structures. The best homology models were structurally optimized using the KoBaMIN server (Rodrigues et al., 2012), which refines either a single protein structure or an ensemble of knowledge-based potential proteins derived from structures deposited in the PDB. The 3D structures were imported into the H++ server (Anandakrishnan et al., 2012) and the protonation states of their residues estimated at neutral pH (7.4 ± 1.0). Reliability of the models was evaluated using MolProbity (Chen et al., 2010).

#### Ligand Preparation

Predicted drugs were standardized using the protocol established by Fourches et al. (2016). For each drug, 2,000 conformations were generated using OMEGA v.3.0.0.1 software (OpenEye Scientific Software, 2019), protonation states were assessed at neutral pH (7.4 ± 1.0) and AM1-BCC charges (Jakalian et al., 2002) were estimated using QUACPAC v.1.7.0.2 (OpenEye Scientific Software, 2013).

#### Molecular Docking

The prepared proteins were subjected to the grid-generation protocol using a molecular probe available in the OEDocking suite v.3.2.0 (OpenEye Scientific Software, 2017) for detection of binding pockets. Grid details (*x*, *y*, and *z* coordinates and box volume) are available in Supplementary Table S1. Molecular docking calculations were performed using the high-resolution protocol of the FRED program with the ChemGauss4 score function (McGann, 2012), in the OEDocking suite. Based on docking scores, a set of structurally diverse drugs were experimentally evaluated *in vitro*.

### Experimental Procedures

#### Chemicals

The following drugs were purchased from commercial chemical databases: dexlansoprazole, mebendazole, albendazole, vistusertib, dactolisib, BGT-226, bifonazole, sertaconazole, butoconazole, luliconazole, midostaurin, raltitrexed, ENMD-2076, and tozasertib (Table 1). All drugs had purity ≥95%. Amphotericin B was purchased from Sigma-Aldrich^®^and used as a positive control. All compounds were solubilized in 5% of the dimethyl sulfoxide (DMSO).

**Table 1:** *In vitro* anti-*Paracoccidioides* spp. activity of the prioritized drugs.

Drug	Predicted *Paracoccidioides* target	Sequence identity^a^ (%)	Docking score	MIC (μM)			MFC (μM)		
				*Pb01*	*Pb03*	*Pb18*	*Pb01*	*Pb03*	*Pb18*
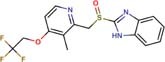 Dexlansoprazole	Na^+^/K^+^-exchanging ATPase alpha chain	35	−10.10	42.3	21.1	169.2	169.2	84.6	338.4
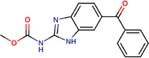 Mebendazole	Tubulin beta chain	84	−9.03	26.4	3.3	13.2	26.4	13.2	26.4
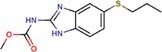 Albendazole	Tubulin beta chain	84	−8.12	58.9	58.9	29.4	235.6	58.9	58.8
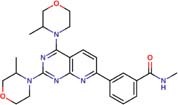 Vistusertib	Phosphatidylinositol 3-kinase TOR2	46	−13.22	1.0	1.0	4.2	1.0	1.0	4.2
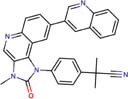 Dactolisib	Phosphatidylinositol 3-kinase TOR2	46	−14.27	66.6	33.3	8.3	266.2	66.6	33.2
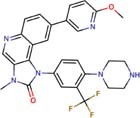 BGT-226	Phosphatidylinositol 3-kinase TOR2	46	−13.03	3.6	3.6	7.3	7.3	7.3	14.5
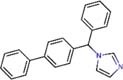 Bifonazole	Lanosterol 14-alpha demethylase	72	−18.36	0.8	0.2	0.8	0.8	0.2	0.7
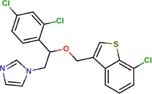 Sertaconazole	Lanosterol 14-alpha demethylase	72	−14.94	0.0036	0.0036	0.0036	0.0150	0.0150	0.0150
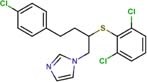 Butoconazole	Lanosterol 14-alpha demethylase	72	−15.73	0.002	0.001	0.002	0.002	0.001	0.002
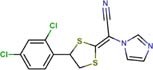 Luliconazole	Lanosterol 14-alpha demethylase	72	−12.36	0.0007	0.0005	0.0007	0.0026	0.0013	0.0026
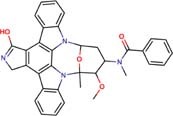 Midostaurin	Protein kinase C	50	−14.51	54.7	13.7	54.7	109.5	27.3	219.0
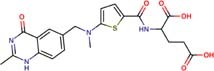 Raltitrexed	Thymidylate synthase	61	−13.32	68.0	68.0	68.0	272.6	272.6	272.6
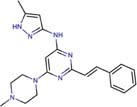 ENMD-2076	Serine/threonine-protein kinase	52	−15.00	14.8	14.8	7.4	14.8	14.8	7.4
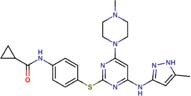 Tozasertib	Serine/threonine-protein kinase	57	−15.11	67.3	16.8	269.1	67.3	33.6	269.01
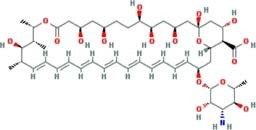 Amphotericin B^∗∗^	Ergosterol	–	–	0.81	0.40	0.81	–	–	–

*^∗, a^sequence identity between predicted Paracoccidioides target and homolog drug target; MFC, minimum fungicidal concentration; MIC, minimum inhibitory concentration. ^∗∗^ Values obtained from the article of Silva et al. (2018).*

#### Microorganism and Culture Conditions

*Pb*01, *Pb*03, and *Pb*18 were incubated in liquid Fava-Netto medium [0.3% protease peptone, 1% peptone, 0.5% (w/v) meat extract, 0.5% (w/v) yeast extract, 1% brain heart infusion, 4% glucose, 0.5% NaCl, 5 μg/mL gentamycin], pH 7.2, for 48 h at 37°C with shaking. Cells were washed with phosphate buffered saline [0.09% Na_2_HPO_4_, 0.02% KH_2_PO_4_, 0.8% NaCl, 0.02% KCl, pH 7.2], transferred to chemically defined medium RPMI 1640 (Sigma-Aldrich^®^) and incubated for 16 h at 37°C with shaking to allow the fungal cells to adapt.

#### Determination of the Minimum Inhibitory Concentration (MIC)

MIC values were determined was Clinical and Laboratory Standards Institute (Clinical Laboratory Standards Institute, 2008) recommendations adapted according to de Paula e Silva et al. (2013). Dilutions of test compounds were added in each well of the microplate in RPMI-1640 with the fungal suspension to a final concentration of 1 × 10^5^ cells/mL. The plates were maintained at 36°C under agitation for 48 h, 20 μL of the 0.02% resazurin solution added, and the incubation continued for 24 h. The MIC was determined by reading the absorbance at 640 and 530 nm.

#### Determination of the Minimum Fungicidal Concentration (MFC)

*Pb*01, *Pb*18, and *Pb*03 cells were exposed to the same concentration of the target drugs and the culture conditions used for the MIC test. From each well, 20 μL of culture was transferred to solidified Fava-Netto medium (Silva et al., 2018). The plates were incubated at 37°C for 7 days. The MFC was defined as the lowest drug concentration at which no fungal growth was visualized.

## Results

### Computational Chemogenomics Approach

**FIGURE 2:**
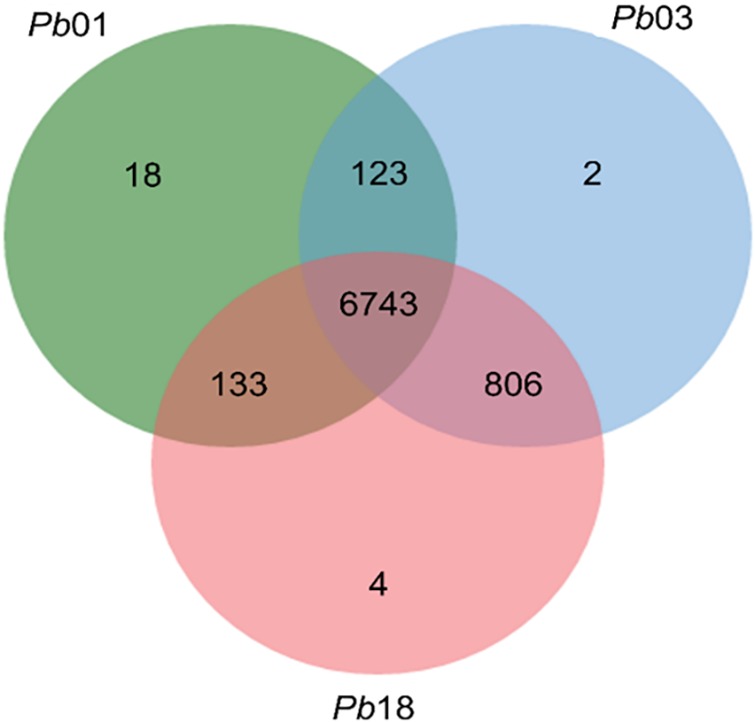
Comparison of the sequences of the three isolates. Venn diagram showing the number of clusters, and the group generated after the comparison. In total, 6782 orthologous proteins were identified in the three isolates.

We developed a computational chemogenomics framework (Figure 1) to repurpose drugs as anti-PCM bioactive using a genome-wide phylogenetic analysis of *Pb*01, *Pb*03 and *Pb*18 isolates. These genomes range from 29.1 to 32.9 Mb and encode 7,610 to 8,130 genes (Desjardins et al., 2011). We identified 6,743 clusters encoding conserved proteins among the three genomes (Figure 2). Each of these protein from *Pb*01 was then used to interrogate two different publicly available databases, DrugBank (Law et al., 2014) and TTD (Li et al., 2018), which provide detailed information about drugs and their targets. This strategy identified 146 potential fungal targets (∼2.15% of the interrogated targets) that might interact with 810 approved drugs or drug candidates in clinical trials.

To investigate which targets might cause *Paracoccidioides* inviability or lethality, a pool of essential *S. cerevisiae* proteins (Zhang, 2004; Zhang and Lin, 2009) was compared with the *Paracoccidioides* targets. Essentiality was inferred by selecting *Pb*01 targets orthologous (*e*-value ≤ 10^−20^) to these *S. cerevisiae* proteins. This strategy resulted in a list of 46 potential druggable targets (31.5% of the interrogated targets) that might interact with 300 approved drugs or drug candidates in clinical trials. Detailed information about the predicted targets and their associated drugs is provided in Table 1 and Supplementary Table S1.

### Homology Modeling

The 3D structures of *Pb*01 targets were not available on the protein data bank (PDB) (Rose et al., 2015) at the time this work was conducted. Homology models were built by aligning the *Pb*01 primary sequences with similar experimentally determined X-ray structures, used as templates. The details of selected templates and homology modeling statistical results are presented in Supplementary Table S1. Validation of the 3D models was done for various levels of structural organization. Statistical analysis of the modeled protein structures showed that most amino acids are within the favored Ramachandran regions (91.60-100%) and have good rotamers (93.98-98.19%), which indicates good quality of the backbone dihedral angles (ψ against φ) and side-chain angles (χ) of amino acids. In addition, acceptable Clashscores (23.13-6.91) and MolProbity scores (1.38-2.35) were obtained for these structures. The Clashscore is the number of serious steric clashes per 1000 atoms. The MolProbity score is a log-weighted combination of the percentage of bad side-chain rotamers, percentage of Ramachandran outliers, and Clashscore, resulting in a number that reflects the resolution of X-ray structures at which those values would be expected (Chen et al., 2010). The overall stereochemistry and conformation characteristics suggest that homology models can be used in prospective molecular modeling investigations.

### Molecular Docking

Virtual screening was carried out using molecular docking to investigate which proteins in the *Pb*01 isolate could interact with drugs available in DrugBank and TTD. Supplementary Table S1 shows 137 drugs have a considerable affinity (ChemGauss4 scores lower than −10.00) with 15 associated *Paracoccidioides* targets. We selected 14 drugs or drug candidates (see Table 1) for prospective analysis according to following characteristics: (a) drugs with lower docking scores; (b) drugs with different chemical scaffolds; and (c) drugs associated with different *Paracoccidioides* targets (see Table 1).

### Experimental Evaluation of Selected Drugs on *Paracoccidioides* spp.

The fourteen drugs were purchased and evaluated *in vitro* against *Pb*01, *Pb*03, and *Pb*18 isolates (Table 1). Six were confirmed to be active against most of the isolates with MICs values <10 μM. Two antineoplasic drug candidates, vistusertib (*Pb*01 MIC = 1.0 μM, *Pb*03 MIC = 1.0 μM, and *Pb*18 MIC = 4.2 μM) and BGT-226 (*Pb*01 MIC = 3.6 μM, *Pb*03 MIC = 3.6 μM, and *Pb*18 MIC = 7.3 μM), showed promising activity profiles against the three isolates. These drugs may be anti-PCM agents because of their lower docking scores with phosphatidylinositol 3-kinase TOR2 of *Pb*01 (*Pb*TOR2). Three antifungal azoles (luliconazole, butoconazole, and sertaconazole) showed activity at nanomolar concentrations (MICs ∼0.002 μM) and were predicted to inhibit lanosterol 14-alpha demethylase of *Pb*01 (*Pb*CYP51). The azole antifungal (bifonazole) was ∼100-400 fold less potent against most of the fungal isolates. All compounds were analyzed for their fungicidal activity as shown in Table 1. Vistusertib, Bifonazole, ENMD-2076 and Tozasertib were fungicidal at their MIC.

## Discussion

Drug repositioning promises a shorter route to the clinic because early stages of drug discovery projects (i.e., hit identification, hit-to-lead and lead optimization, preclinical studies, bulk manufacturing, and even phase I clinical trials) have, in many cases, already been completed and can be bypassed. Drug repositioning can reduce the risk, cost and the timeline to market and could provide strategic advantages by introducing new treatments against PCM (Ashburn and Thor, 2004; Chong and Sullivan, 2007; Novac, 2013; Sbaraglini et al., 2016). Despite a potential for rapid clinical impact, a systematic effort has yet to identify new anti-PCM drugs. We have developed a computational chemogenomics framework to identify new anti-PCM drugs using the assumption that homologous proteins have enhanced probability of sharing the same ligands (Andrade et al., 2018). In contrast to traditional drug repurposing approaches, that focus on specific proteins, our chemogenomics framework identifies potential drugs based on the entire *Paracoccidioides* genome. Some concerns have been raised about using fungal targets with orthologs in humans, in order to avoid and adverse effects. However, our approach has promise because orthology provides evidence of druggability and offers potential scaffolds. Drug selectivity is predicted and can be optimized using structural analogs or bioisosteres designed to interact more efficiently with the target instead of homologs in humans. Consequently, an initial difficulty in drug repurposing can become opportunity (Beghyn et al., 2011; Njoroge et al., 2014).

Our computational chemogenomics framework allowed prioritization of 14 drugs for experimental validation against three *Paracoccidioides* isolates (Table 1). Two drug candidates (BGT-226 and vistusertib) predicted to be inhibitors of *Pb*TOR2 showed antifungal activity at low micromolar concentrations. This provided confidence that our strategy for predicting new anti-PCM drugs is useful. TOR2 mediates two essential functions in fungi: (a) protein synthesis and cell cycle progression and (b) the cell-cycle-dependent organization of the actin cytoskeleton. Cells lacking TOR2 do not display a G0 arrest phenotype but instead undergo a few cell divisions before arresting randomly in the cell cycle (Kunz et al., 1993). Yeast mutants defective in TOR2 do not exhibit the normal polarized distribution of the actin cytoskeleton and are rescued by overexpression of TCP20, an actin-specific chaperone (Schmidt et al., 1996). Importantly, overexpression of a TOR1 homolog does not suppress the loss of TOR2 in fungi (Helliwell et al., 1994). Therefore, *Pb*TOR2 may prove to be an attractive drug target for anti-PCM drug discovery projects upon phenotypic/pharmacological validation.

**FIGURE 3:**
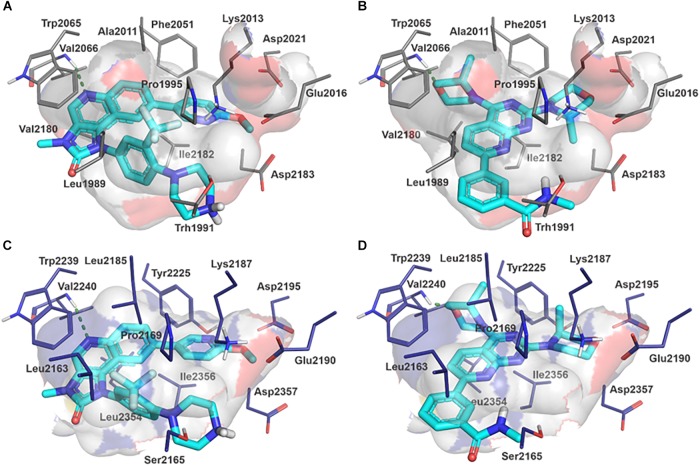
Predicted intermolecular interactions of BGT-226 **(A,C)** and vistusertib **(B,D)** with ATP binding sites of *Pb*TOR2 (amino acid residues in gray) and human TOR2 (amino acid residues in blue).

Molecular docking studies allowed us to rationalize the interactions of vistusertib and BGT-226 with *Pb*TOR2 and design more potent and selective analogs (Figure 3). A common feature of BGT-226 and vistusertib scaffolds is their ability to bind to the adenosine triphosphate (ATP) pocket and make a hydrogen bond with the backbone residues of the hinge (Roskoski, 2016).

Figure 3A shows the pyridine ring of BGT-226 can form a hydrogen bond (represented as a green dashed lines) with the amine backbone of the Val2066 and a π-stacking interaction with Trp2065. In addition, the (trifluoromethyl) benzene moiety of BGT-226 can interact with a hydrophobic pocket formed by Pro1995, Ala2011, Leu1989, and Thr1991 while the phenyl and 2-methoxypyridine can interact with the pocket formed by Val2180, Phe2051, and Ile2181. Analysis of vistusertib binding mode (Figure 3B) showed that the morpholine ring can form a hydrogen bond (represented as green dashed lines) with the amine backbone of Val2066 while the phenyl and pyridine can interact with the hydrophobic pocket composed of Pro1995, Ala2011, Leu1989, and Ile2182. Similar interactions and affinities were also observed for BGT-226 (Figure 3C) and vistusertib (Figure 3D) in the ATP binding site of the human homolog TOR2. Despite similar binding modes and affinities with the human TOR2, there are considerable differences in hydrophobicity of the two binding sites. For instance, amino acid residues of the binding site for fungal TOR2 (e.g., Phe2051 and Ala2011) were substituted in human TOR2 (e.g., Tyr2225 and Leu2185) proteins. It therefore appears that the binding site of *Pb*TOR2 can accommodate bulkier ligands. These structural differences may help the design more potent and selective anti-PCM lead candidates by optimizing ligand interactions in *Pb*TOR2 binding site. Prospective studies will also include multiparametric optimization of the pharmacokinetics and toxicological properties of BGT-226 and vistusertib analogs using quantitative structure-property relationships using deep learning (Goh et al., 2017; Jing et al., 2018).

Another drug validated *in vitro* was mebendazole, which showed activity against *Pb*03 isolate (Table 1). Mebendazole is an anti-helminthic drug predicted as an inhibitor of the tubulin beta chain, an important protein for the formation of microtubules, and an essential element of the cytoskeleton of eukaryotic cells (Janke, 2014). Used for the treatment of infections caused by parasitic worms, mebendazole has also been studied as a possible treatment of mycosis caused by *Cryptococcus neoformans* (Joffe et al., 2017). Thus, structural modifications that increase the activity of mebendazole against different species of *Paracoccidioides* are of interest.

*In vitro* assays also indicated that antifungal azoles as sertaconazole, butaconazole and luliconazole gave MICs and MFCs at nanomolar concentrations and were more potent than control drug amphotericin B (Table 1). The azoles antifungals are inhibitors of CYP51, an essential enzyme that catalyzes the demethylation of lanosterol to ergosterol (Sagatova et al., 2015). The latter is involved in maintaining membrane integrity. Despite their potency and target essentiality, none of these antifungal drugs shows the needed bioavailability to treat systemic infections. Therefore, new formulations will be needed to overcome pharmacokinetic issues for a viable PCM treatment.

## Conclusion

We developed a computational chemogenomics framework that allowed prioritization of 14 potential anti-PCM drugs for experimental validation against three *Paracoccidioides* isolates. Two anti-cancer drug candidates (BGT-226 and vistusertib) predicted to be inhibitors of *Pb*TOR2 showed potent antifungal activity at low micromolar concentrations. Although BGT-226 and vistusertib have not been tested against *Pb*TOR2, docking studies suggest that they likely have a mechanism of action involving fungal TOR2. We also evaluated the anti-PCM activity of four antifungal azoles (bifonazole, sertaconazole, butoconazole, and luliconazole) predicted to be inhibitors of *Pb*CYP51. The *in vitro* assays indicate that three of these azoles have MICs and MFCs in the nanomolar range. We recognize that such compounds may be unsuitable in the clinic due to limited potency and/or pharmacokinetic properties. Therefore, we recommend prospective lead optimization studies for BGT-226 and vistusertib. In addition, we recommend the development of new pharmaceutical formulations to improve oral bioavailability of the putative CYP51 inhibitors and structural modification studies to improve the activity of mebendazole. We suggest the computational chemogenomics approach to drug repurposing we used has the potential to address the urgent need to discover new antifungals active against PCM.

## Data Availability

The raw data supporting the conclusions of this manuscript will be made available by the authors, without undue reservation, to any qualified researcher.

## Author Contributions

BN designed the computational framework. BN and AdO performed the computational experiments. AdO and LS performed the experimental assays. BN, MP, CS, and CA analyzed the data and wrote the manuscript. All authors have reviewed, discussed, edited, and approved the final manuscript.

## Conflict of Interest Statement

The authors declare that the research was conducted in the absence of any commercial or financial relationships that could be construed as a potential conflict of interest.
